# PEG-Free Polyion Complex Nanocarriers for Brain-Derived Neurotrophic Factor

**DOI:** 10.3390/pharmaceutics14071391

**Published:** 2022-06-30

**Authors:** James M. Fay, Chaemin Lim, Anna Finkelstein, Elena V. Batrakova, Alexander V. Kabanov

**Affiliations:** 1 Center for Nanotechnology in Drug Delivery, Eshelman School of Pharmacy, University of North Carolina, Chapel Hill, NC 27599-7362, USA; jfay@med.unc.edu (J.M.F.); chaemin@email.unc.edu (C.L.); batrakova@unc.edu (E.V.B.); 2 Department of Biochemistry and Biophysics, School of Medicine, University of North Carolina, Chapel Hill, NC 27599-7260, USA; 3 Division of Pharmacoengineering and Molecular Pharmaceutics, Eshelman School of Pharmacy, University of North Carolina, Chapel Hill, NC 27599-7260, USA; 4 Laboratory of Chemical Design of Bionanomaterials, Faculty of Chemistry, M.V. Lomonosov Moscow State University, 119992 Moscow, Russia

**Keywords:** poly(ethylene glycol), poly(2-oxazoline), polyion complex, nanoformulation, brain-derived neurotrophic factor, microfluidic mixing

## Abstract

Many therapeutic formulations incorporate poly(ethylene glycol) (PEG) as a stealth component to minimize early clearance. However, PEG is immunogenic and susceptible to accelerated clearance after multiple administrations. Here, we present two novel reformulations of a polyion complex (PIC), originally composed of poly(ethylene glycol)_113_-b-poly(glutamic acid)_50_ (PEG-PLE) and brain-derived neurotrophic factor (BDNF), termed Nano-BDNF (Nano-BDNF PEG-PLE). We replace the PEG based block copolymer with two new polymers, poly(sarcosine)_127_-b-poly(glutamic acid)_50_ (PSR-PLE) and poly(methyl-2-oxazolines)_38_-*b*-poly(oxazolepropanoic acid)_27_-*b*-poly(methyl-2-oxazoline)_38_ (PMeOx-PPaOx-PMeOx), which are driven to association with BDNF via electrostatic interactions and hydrogen bonding to form a PIC. Formulation using a microfluidic mixer yields small and narrowly disperse nanoparticles which associate following similar principles. Additionally, we demonstrate that encapsulation does not inhibit access by the receptor kinase, which affects BDNF’s physiologic benefits. Finally, we investigate the formation of nascent nanoparticles through a series of characterization experiments and isothermal titration experiments which show the effects of pH in the context of particle self-assembly. Our findings indicate that thoughtful reformulation of PEG based, therapeutic PICs with non-PEG alternatives can be accomplished without compromising the self-assembly of the PIC.

## 1. Introduction

Nanoformulations incorporating poly(ethylene glycol) (PEG) coronas have enabled immune evasion and the subsequent delivery of packaged therapeutic agents [1,2] However, despite PEG’s status as the “gold standard” stealth polymer, several issues have been identified. Accelerated clearance after repeat administration of stealth nanoparticles due to elicitation of antibodies has long been noted in the literature [3]. Furthermore, possibly due to the ubiquity of PEG in pharmaceutical products and consumer goods, much of the population has existing α-PEG antibodies [4], which could yield accelerated clearance of PEG based pharmaceutical agents or allergic reactions to critical therapies. Recent reports of possible allergic reactions to PEG in the Pfizer and Moderna vaccines against SARS-COVID-2 have brought an already expansive body of work to the forefront of the scientific consciousness [5,6,7,8,9]. PEG’s prevalence in modern therapeutics and immunogenicity can combine to create a population level problem as an increasing number of widely distributed therapies incorporate PEG.

Many groups, our own included, have presented a number of novel nanoformulations whereby small molecules, nucleic acids, and proteins are protected by PEG coronas. In one particular example, we presented a polyion complex (PIC) composed of poly(ethylene glycol)_113_-b-poly(glutamic acid)_50_ (PEG-PLE) and brain-derived neurotrophic factor (BDNF), termed Nano-BDNF PEG-PLE, as a therapeutic candidate for the treatment of neurodegenerative diseases [10,11]. BDNF has demonstrated great promise in treating animal models of several diseases including ischemic stroke [10], Alzheimer’s disease [12], Parkinson’s disease [11,13], etc. [14]. However, delivery of BDNF to the brain or across the blood–brain barrier (BBB) remains difficult, despite BDNF’s ubiquity in the body. Endogenous trans-BBB transporters are quickly saturated, serum half life is short, and direct administration of BDNF to the brain is attenuated by rapid efflux [11,15]. Nano-BDNF PEG-PLE nanoparticles effectively mediated the delivery of BDNF to its target receptor after intranasal to brain administration [11]. Additionally, we have demonstrated that Nano-BDNF PEG-PLE presents clear benefits over unformulated BDNF in the treatment of Parkinson’s disease and ischemic stroke models in mice while preserving BDNF’s circulation and residence in affected areas [10,11].

PEG is a long, flexible, and hydrophilic polymer which can comprise a corona which entropically exclude opsonins from penetrating into the nanoparticle and interacting with the therapeutic cargo [16,17,18]. Similar polymers have shown the same stealth effect, allowing nanoparticles to circulate longer and minimizing unwanted clearance or surface fouling [19,20,21]. Work from our own lab and others has identified a variety of polymers, which recapitulate the long, hydrophilic, and flexible nature of PEG [19,22,23,24,25,26,27]. In particular, our group has extensive experience utilizing poly(2-oxazolines), which present a useful platform for the production of functionalized block copolymers [19,28,29,30,31,32,33,34]. Convenient extension of polymers using living-cationic-ring-opening-polymerization (LCROP) enables precise control of block length and organization. Several advancements using polyoxazoline constructs to form drug carrying nanoparticles which demonstrate a stealth effect similar to PEGylated liposomes or PICs have been presented [19,24,29,30,31,32,33,34,35,36,37]. Similarly, other groups have used poly(sarcosine) (PSR), another flexible and hydrophilic polymer, to similar effect in non-fouling surfaces and stealth nanoparticles [20,26,38,39,40,41,42].

Here, we introduce two types of PIC’s similar to the original Nano-BDNF PEG-PLE but free of PEG. A nanoparticle composed of BDNF and poly(sarcosine)_127_-b-poly(glutamic acid)_50_ (Nano-BDNF PSR-PLE) and a series of nanoparticles composed of BDNF and poly(methyl-2-oxazolines)_x_-*b*-poly(oxazolepropanoic acid)_y_-*b*-poly(methyl-2-oxazoline)_z_ (PMeOx_x_-PpaOx_y_-PmeOx_z_) (B1-5) (Nano-BDNF B1-5) are presented. We examine the formulation of several Nano-BDNF species and present two nanoparticles, which are small and narrowly dispersed. We also pursue a deeper understanding of the thermodynamics of association between BDNF and our various polymers as well as their behavior to demonstrate similarity between our three chosen polymers. Finally, we consider the application of the nanoparticles to cells stably transfected with tropomyosin receptor kinase B (TrkB), the primary receptor for BDNF [43], in order to assure that we do not prohibit activation of the desired target. In conclusion, we present two novel non-PEG formulations, which demonstrate similarity to the original Nano-BDNF PEG-PLE formulation in morphology, activity, and thermodynamic behavior with potential for therapeutic effect in neurological diseases.

## 2. Materials and Methods

### 2.1. Materials

Methyl triflate, 2-methyl-2-oxazoline, methoxycarboxyethyl-2-oxazoline, and piperidine were obtained from Sigma Aldrich. Dialysis membrane with molecular weight cut off [MWCO] 3500 was purchased from Spectra/Pore. The following materials were purchased from Thermo/Fisher: WhatMan Anotop 0.025 μm non-sterile filters, 25 gauge 0.5 inch, long blunt needle (Strategic Applications Inc., Lake Villa, IL, USA), 40% acrylamide 29:1, TEMED, Ammonium persulphate, Malvern Folded Cap Cells, DLS cuvettes (Malvern Pananalytical, Malvern, UK), radioimmunoprecipitation assay buffer, HALT protease and phosphatase inhibitor cocktail, SuperSignal™ West Pico PLUS Chemiluminescent Substrate, iBRIGHT prestained protein ladder, Geneticin™ Selective Antibiotic (G418 Sulfate), Neurobasal Media, B27 supplement, Glutamax 100× acetonitrile, potassium carbonate, diethyl ether, methanol, agarose tablets, and sodium hydroxide. BDNF was obtained from Novoprotein, which later became Bon Opus Biosciences. PSR-PLE was obtained from Peptide Solutions LLC. Colorado calf serum was obtained from the Colorado Serum Company. We obtained Bio-Safe Coomassie Stain and Native Sample buffer for protein gels 4× from Bio-Rad, Hercules, CA, USA.

### 2.2. Polymer Synthesis

Under dry and inert conditions achieved in MBraun glovebox with N2 atmosphere, 1 molar equivalent of the polymerization initiator, methyl triflate (MeOTf), and 10 or 30 molar equivalents of 2-methyl-2-oxazoline (MeOx) were dissolved in 2 mL of acetonitrile (ACN). The mixture was sealed and moved to react in a microwave for 15 min at 150 W and 130 °C. After cooling, the solution was returned to dry and inert conditions where 10 or 30 molar equivalents of the monomer for the second block, methoxycarboxyethyl-2-oxazoline (MestOx), were added. The mixture was sealed and moved to the microwave following the same procedure as above. After cooling, the procedure was repeated with 10 or 30 molar equivalents of MeOx. Polymerization was terminated by adding 3 molar equivalents of piperidine and microwaving for 1 h at 150 W and 40 °C. An excess of K_2_CO_3_ was added to ensure complete cessation of the reaction. The solution was stirred overnight, filtered through a 0.45 μm filter, precipitated in diethyl ether, collected, and freeze dried. The product was dissolved in methanol and 0.1 N NaOH was added to 1 molar equivalent NaOH with the methyl ester group on the poly(MestOx) block of the copolymer to convert to PPaOx. The resultant solution was allowed to react for 2 h at 80 °C. The solution was then dialyzed against distilled water using a dialysis membrane (Spectra/Pore; molecular weight cut off (MWCO) 3500), product was freeze dried for storage until use.

### 2.3. Polymer Characterization by ^1^H NMR

Copolymers were suspended in deuterated methanol (MeOD) and ^1^H nuclear magnetic resonance (NMR) spectra were obtained using an INOVA 400 NMR machine. Data were analyzed using MestReNova (11.0). Spectra were calibrated using the MeOD solvent signal at 4.78 ppm. The number average molecular weight was determined by calculating the ratio of the initiator and each repeating unit.

### 2.4. Preparation of PIC by Manual Mixing

The formulation was manufactured as described previously in Jiang et al. [11] Briefly, a concentrated solution of a block copolymer (0.1, 1, or 10 mg/mL) in a specified buffer was added dropwise to the BDNF solution ([BDNF] = 1 mg/mL); each drop was interspersed with a gentle vortex for 10 s. The solution was then diluted to the desired concentration using the buffer and vortexed for another 30 s, after which the solution was incubated for 30 min at room temperature.

### 2.5. Preparation of PIC Using a Microfluidic Mixer

Separate solutions of BDNF and copolymer in a specified buffer were prepared at 2× the desired end concentrations. We used either phosphate buffered saline (PBS) (10 mM NaH_2_HPO_4_, 1.8 mM KH_2_PO_4_, 2/7 mM KCl, 137 mM NaCl, pH 7.4)) or lactate buffered Ringer’s solution (LR) (100 mM NaCl, 3.7 mM KCl, 1.3 mM CaCl_2_, 1.2 mM MgCl_2_, 28 mM NaHCO_3_, 20 mM NaC_3_H_5_O_3_, pH 7.4). The BDNF and copolymer solutions were loaded into 1 mL syringes which were then connected to the microfluidic mixer apparatus (Figure S1) described previously [11]. The solutions were each injected into the mixer at 50 µL/min to mix at a 1:1 volume ratio. The first 100 μL were discarded to allow only properly mixed samples to be collected before experiments.

### 2.6. Nanoparticle Size, Dispersity, and Zeta Potential

Nano-BDNF was prepared at the desired Z_−/+_ ratio. The particle size and polydispersity index (PDI) were analyzed by dynamic light scattering (DLS) using the Zetasizer Nano ZS (Malvern Instruments Ltd., Malvern, UK) with a scattering angle of 173° at RT. For all DLS measurements, the BDNF concentration was 0.05 mg/mL. Unless specified otherwise, the DLS sizes are reported as intensity weighted (z-averaged) diameters. The nanoparticle tracking analysis (NTA) was performed using the NanoSight NS500 (Malvern Panalytical) or Zetaview (ParticleMetrix). All samples were prepared at BDNF concentration 0.005 mg/mL. For the measurements of particle size and distribution using the NanoSight NS500, the samples were stored on ice and allowed to return to RT prior to use. Readings were taken under flow for 60 s at 20 µL/s using the syringe pump. For the measurements of particle size, distribution, and zeta potential using the Zetaview, the samples were prepared at BDNF concentration 0.005 mg/mL and analyzed either immediately or incubated at RT for various times. Before the measurements, these samples were diluted 10X. Both NanoSight NS500 and Zetaview are reported as number-weighted diameters.

### 2.7. Transmission Electron Microscopy (TEM)

Samples for TEM were prepared using the microfluidic mixer at Z_−/+_ = 5 in 10 mM phosphate, pH 7.4. Images were taken using a Thermofisher Talos F200X at an accelerating voltage of 200 kV in TEM mode. Samples were drop cast on a TEM formvar carbon grid then stained using aqueous 2% Uranyl Acetate solution. Image processing was performed using Velox, then analyzed using ImageJ.

### 2.8. Horizontal Agarose Gel Electrophoresis (HAGE)

Agarose gels (0.5% wt.) were prepared using 20 mM MOPS buffer, containing 2 mM sodium acetate, pH 7.4, which was also used as the running buffer. Nano-BDNF was manually prepared as described above in LR solution, at a concentration of 0.075 mg/mL and volume of 18 μL. The sample was diluted 2× with Native Loading Buffer, pH 6.8 (Bio-Rad) and 35 μL of the final solution were loaded into the wells. Electrophoresis was performed with a constant voltage of 80 V for 1 h. The gel was washed in DI water 2× and stained using Biosafe Coomassie Stain (Bio-Rad) for at least 1 h. Destain was performed using DI water until the gel was mostly clear. Images were taken using the FluorChem E machine (ProteinSimple).

### 2.9. Isothermal Titration Calorimetry (ITC)

BDNF and polymer solutions were dialyzed against 10 mM phosphate, pH 7.4, 10 mM phosphate, pH 5.8, or 10 mM sodium acetate, pH 5.0 overnight with at least two buffer changes. BDNF was diluted to 10 µM and the polymers were diluted to the indicated concentrations then stored on ice. Samples were loaded into the MicroCal PEAQ-ITC machine (Malvern Panalytical) and stored at 4 °C in the tray. The cell and syringe were kept at 25 °C and the experiment was performed at 25 °C. The polymer was added in 2 μL steps except for the first addition which was 0.2 μL. The cell was allowed to incubate for 3 min between each step. Data analysis was performed using the MicroCal ITC analysis software in Origin.

### 2.10. Potentiometric Titration of Polymers

Polymers were solubilized in 10 mL of nitrogen flushed dH_2_O and a 1.1 molar equivalent aliquot of 1.1 M HCl was added to the 10 mL of polymer solution at a concentration of 500 μM ionizable units. 50 mM NaOH was added in 10 μL increments, the pH was allowed to stabilize (~1 min dwell time), and readings were taken using an Oaklon ion 700 pH meter with Fisherbrand™ accumet™ glass electrode.

### 2.11. Cell Culture

NIH 3T3 cells stably transfected with TrkB (NIH 3T3 TrkB) were cultured in GlutaMax DMEM supplemented with 10% calf serum, G418 (100 μg/mL), penicillin (100 μg/mL), and streptomycin (100 μg/mL). Cells were cultured at 37 °C in a humidified CO_2_ (5%) incubator.

### 2.12. BDNF Stimulation

The NIH 3T3 TrkB cells were plated at 5 × 10^5^ cells/well on 6-well plates 24 h before treatment. Five hundred nanograms of BDNF or equivalent amount of Nano-BDNF was added to the wells then incubated for 5 min at 37 °C in the incubator. Formulations were prepared via microfluidic mixing at Z_−/+_ = 5 in cell culture media. Media was removed and replaced with radioimmunoprecipitation assay buffer with HALT™ proteinase and phosphatase inhibitors (ThermoFisher, Waltham, MA, USA), then the plate was moved to 4 °C and agitated using a plate shaker. Material was collected and separated into supernatant and pellet. The supernatant was measured for protein concentration by the BCA assay, then 25 μg of protein was loaded into each well and electrophoresed through a 12.5% SDS-PAGE gel, transferred to PVDF membrane, and visualized via horseradish peroxidase (Supersignal^TM^ West Pico Plus Chemiluminescent Substrate) following standard Western blotting procedures. The following antibodies were used to visualize their cognate proteins: Phospho-p44/42 MAPK (Erk1/2) (Thr202/Tyr204) Antibody (CST 9101), p44/42 MAPK (Erk1/2) Antibody (CST 9102), and Anti-Rabbit HRP (CST 7074S).

## 3. Results

### 3.1. Manufacture and Characterization of Poly(2-oxazoline) Block Copolymers

**Figure 1:**
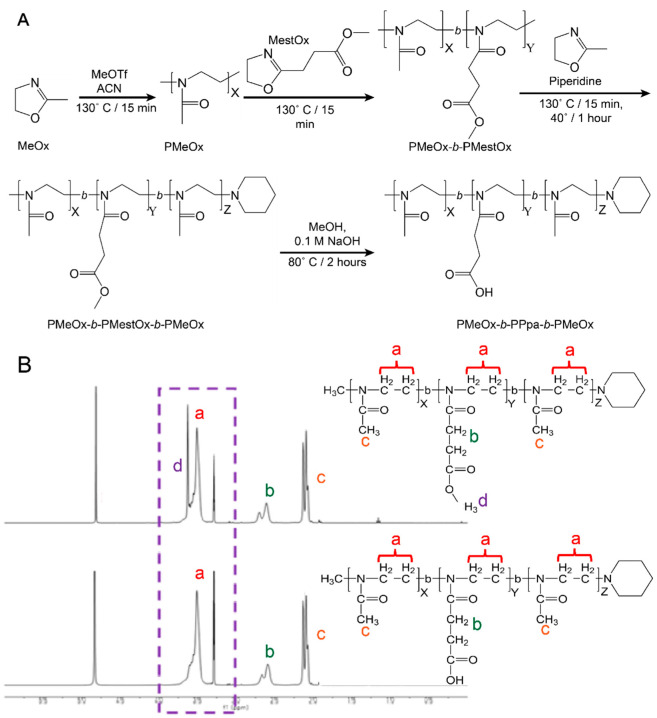
Polymer synthesis and characterization**.** (**A**) Synthesis scheme for the preparation of poly(2-oxazoline) tri-block copolymers via LCROP. Briefly, the initiator, MeOTf, was reacted with MeOx in ACN for 15 min at 130 °C followed by sequential polymerization of subsequent blocks in ACN for 15 min each then termination with piperidine reacted for 1 h at 40 °C. The protective methyl group on the MestOx block was removed by reacting with NaOH in MeOH for 2 h at 80 °C. (**B**) ^1^H-NMR peak assignments of PMeOx-*b*-MestOx-*b*-PMeOx and PMeOx-*b*-PPaOx-*b*-PMeOx. Note the elimination of peak (d) representing the deprotection of the hydroxyl group on the middle block. Peaks (a, b, and c) present no change during deprotection. Spectra of the four polymers produced can be found in the supplement (Figure S2).

**Figure 2:**
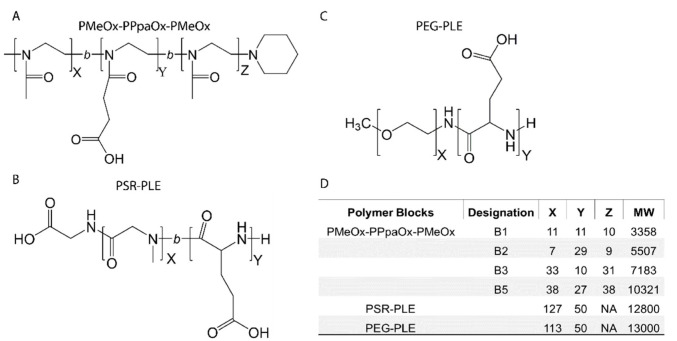
Block copolymer structures (**A**–**C**) and molecular characteristics (**D**).

Previous work demonstrated that BDNF readily associated with the negatively charged PEG-PLE diblock copolymer to form stable nanoparticles [10,11]. Pursuant to our objective of replacing PEG with alternative polymers, we produced four block copolymers using LCROP as described previously [44] (Figure 1). The new polymers were designed as anionic A-B-A type block copolymers composed of hydrophilic uncharged PMeOx (A block) and negatively charged PPaOx (B block). The amounts of monomers, ten or thirty in each step, added during synthesis were selected to produce four variations of PMeOx-PPaOx-PMeOx with the following patterns: short-short-short, short-long-short, long-short-long, and long-long-long (Figure 2). Structure and length ratio were confirmed via ^1^H-NMR (Figure 1, Supplementary Figure S2). As an alternative approach to formulating BDNF, we used an anionic PSR-PLE di-block copolymer that was available commercially with a similar structure to the original PEG-PLE polymer used in our previous study (Figure 2).

### 3.2. Nano-BDNF Candidate Innowing

The length and structure of the copolymer blocks can drastically affect self-assembly of block copolymers with proteins or other cargo for delivery [45]. In addition, the mode of mixing is of paramount importance for formulation of PIC that can form non-equilibrium, slowly transitioning aggregates [22,46]. This is characteristic of PICs that contain proteins or peptides, such as BDNF, which can self-aggregate because of intermolecular hydrophobic interactions or hydrogen bonding. To quickly identify which block copolymers are most suitable for formulation, we adopted a winnowing strategy. Towards that end, we formulated Nano-BDNF in 10 mM Hepes, pH 7.4 to interrogate block copolymer-protein interaction and identify which copolymers yield relatively small, narrowly dispersed particles.

The ionic strength, salt identity, and pH are all expected to strongly affect the formation of the PIC particles. Initial formulation in minimal conditions of only HEPES buffer allows us to identify which copolymers form stable PICs with BDNF in the near absence of salts. We initiated by manually formulating Nano-BDNF in 10 mM HEPES, pH 7.4, by mixing each copolymer and BDNF. In addition to the effects of buffer components, the ratios in which copolymer and protein are mixed may strongly affect the formation of particles in terms of size, dispersity, stability, etc. [11]. The mixing ratio between carboxyl groups of copolymer and amino groups of BDNF is defined here as follows:

(1) $$Z_{-/+}=\frac{\left[\mathrm{Copolymer}\right]\times \left(\mathrm{degree}\;\mathrm{of}\;\mathrm{polymerization}\;\mathrm{of}\;\mathrm{ionic}\;\mathrm{block}\right)}{\left[\mathrm{BDNF}\right]\times \left(44\;\mathrm{positive}\;\mathrm{charges}\right)}.$$

**Figure 3:**
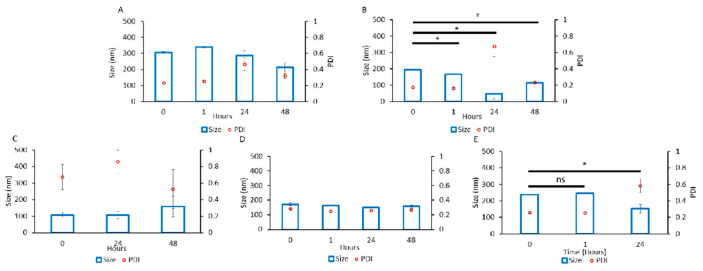
Particle size and dispersity of Nano-BDNF. The complexes were prepared by manual mixing of the solutions of BDNF and (**A**) B1, (**B**) B2, (**C**) B3, (**D**) B5, or (**E**) PSR-PLE in 10 mM HEPES, pH 7.4. The component charge ratio was Z_−/+_ = 1 (**B**–**E**) or Z_−/+_ = 2 (**A**). The DLS measurements were carried out for up to 48 h. Values are mean ± SEM, * *p* < 0.05, unmarked plots do not show statistically significant differences. Statistical significance was established using an unpaired two-tailed Student’s *t*-test.

Each PIC was formulated at several Z_−/+_ ratios (1, 2, 5, 10). We assessed the size and dispersity of the particles using DLS over two days. The poly(2-oxazoline) tri-block copolymers showed major differences in their propensity to associate with BDNF. While we observed particle formation in all cases, copolymers B2 and B5 yielded particle populations that were generally smaller and more uniform (Figure 3 and Supplementary Figure S3–S12). In both cases, the particle sizes were under 200 nm and the PDI was from 0.2 to 0.3 (except for 24 h time point for B2 that show an unexpectedly high PDI). Formulation with B1 formed larger aggregates (200 to 350 nm) and B3 produced highly disperse particles (PDI from 0.5 to 0.8). Therefore, we disregarded those copolymers in future studies and focused on B2 and B5. We noted that the formulations containing B2 and B5 (at several Z_−/+_ ratios, i.e., 2, 5, and 10) were variable and poorly reproducible, indicating the capricious nature of the manual mixing. Nano-BDNF formulated with PSR-PLE was also not greatly amenable to the formulation at this initial stage, as mixing formed highly disperse PIC particles (Figure 3, Supplementary Figure S3). However, we continued optimization using PSR-PLE due to similarity to the structure of PEG-PLE used in Jiang et al. [11].

**Figure 4:**
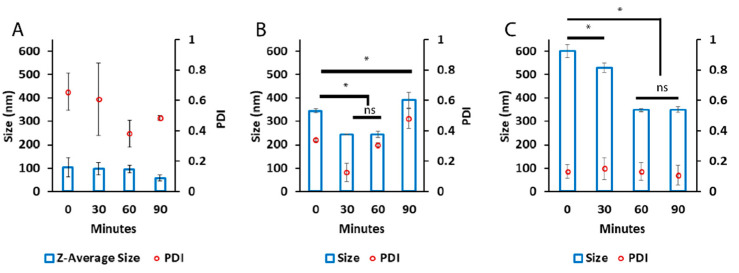
Particle size and dispersity of Nano-BDNF in the presence of small-molecular mass electrolyte. All Nano-BDNF samples were prepared at Z_±_ = 5 by manual mixing of the solutions of BDNF and (**A**) B2, (**B**) B5, or (**C**) PSR-PLE in 10 mM HEPES, pH 7.4, 150 mM NaCl. The DLS measurements were carried out for over 2 h. Values are mean ± SEM, * *p* < 0.05, unmarked plots do not show statistically significant differences. Statistical significance was established using an unpaired two-tailed Student’s *t*-test.

The effects of low molecular-mass electrolytes on PICs have been noted in the literature such that different salts can affect particle formation, size, and dispersity [45]. To assess these effects in our systems, we opted for the simple addition of 150 mM NaCl to a 10 mM HEPES, pH 7.4 solution. Nano-BDNF was prepared using B2, B5, and PSR-PLE via manual mixing. We did not observe a decisive advantage using any one copolymer under these conditions: B2 yielded small particles with very high polydispersity, PSR-PLE yielded very large but narrowly disperse particles, and B5 was a mix (Figure 4). Most notably, the particles formed at higher Z_−/+_ ratios using B5 and PSR-PLE (Figure S13E,F,I) were smaller and less dispersed than in the absence of added salt. It may be that the addition of NaCl allowed ion exchange and facilitated packing. The number weighted particle size for B2 was negligible compared to the intensity weighted particle size, indicating only a small number of aggregates existed in the presence of salt (Figure S14A–C).

**Figure 5:**
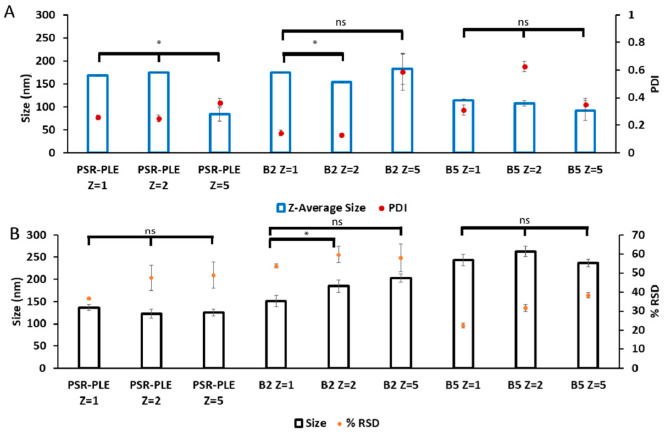
Particle size and dispersity of Nano-BDNF prepared by microfluidic mixing. Nano-BDNF samples were prepared in 10 mM Hepes, pH 7.4 at various Z_−/+_ ratios. The complexes were characterized by (**A**) DLS or (**B**) NTA using Nanosight NS500. Values are mean ± SEM, * *p* < 0.05, unmarked plots do not show statistically significant differences. Statistical significance was established using an unpaired two-tailed Student’s *t*-test.

To select the best copolymers to continue optimization, we altered the formulation methodology to include microfluidic mixing, which has yielded decreased particle size and dispersity in past publications [11,47,48]. B2, B5, and PSR-PLE were incorporated into Nano-BDNF using microfluidic mixing in 10 mM HEPES, pH 7.4 with no elementary salt added. We measured size and dispersity using DLS and NTA (Figure 5). Using microfluidic mixing we generally observed smaller and less disperse particles (Figure 5) when compared to manual formulation in the same buffer (Figure 3 and Supplementary Figures S3–S12). PSR-PLE particles formed using this technique considerably decreased the size and narrowed dispersity compared to manual mixing (Figure 3). This result is consistent with previous work where Nano-BDNF formulated with PEG-PLE yielded smaller, more narrowly disperse particles when formulated using the same microfluidic mixer. Considering the totality of our results (Figure 3, Figure 4 and Figure 5 and Supplementary Figures S3–S14), we elected to pursue formulation with PSR-PLE and B5. These two nanoparticle formulations, similar to the original PEG-PLE-based Nano-BDNF, had a nearly spherical shape as determined by TEM (Supplementary Figure S15).

### 3.3. Preparation of Nano-BDNF Particles Suitable for Injection

**Figure 6:**
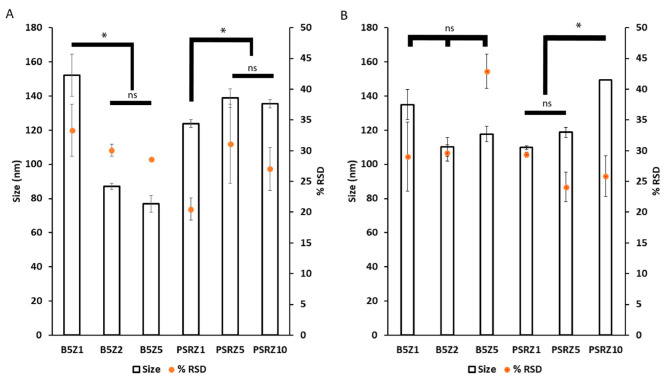
Particle size and distribution of Nano-BDNF samples in isotonic solutions. The complexes were prepared by microfluidic mixing in (**A**) PBS and (**B**) LR. The measurements were carried out within 10 min after mixing at RT. The NTA measurements were carried out using Nanosight NS500. Values are mean ± SEM, * *p* < 0.05, unmarked plots do not show statistically significant differences. Statistical significance was established using an unpaired two-tailed Student’s *t*-test.

The final stage of optimization was to prepare Nano-BDNF in isotonic solutions formulated with saline that are commonly used for in vivo injections. We selected PBS and LR for formulation and prepared Nano-BDNF in these buffers using copolymers B5 and PSR-PLE via microfluidic mixing. We analyzed the resultant particles size and distribution right after preparation (Figure 6) and over time (Figure S16). Right after mixing, B5 formed the smallest, least disperse particle population when formulated in PBS at Z_−/+_ = 5. However, the formulations in the LR were still promising, since Nano-BDNF B5 at Z_−/+_ = 2 and Nano-BDNF PSR-PLE at Z_−/+_ = 5 both yielded particles with sizes less than 120 nm and with RSD under 30. When size and dispersity were measured over time, we found that the LR solution yielded smaller sizes and narrower dispersity for both Nano-BDNF B5 and Nano-BDNF PSR-PLE after several hours of use (Figure S16). Therefore, we used the LR buffer for the next experiments.

### 3.4. HAGE Visualization of Nano-BDNF

**Figure 7:**
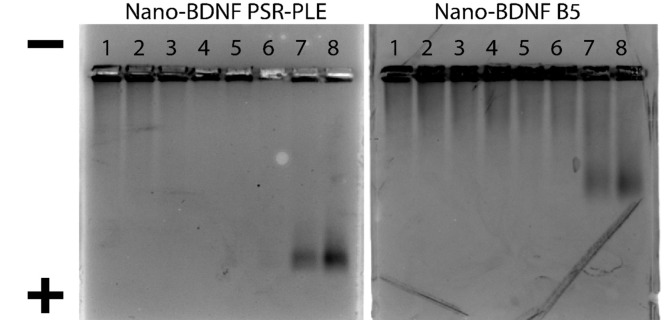
HAGE analysis of Nano-BDNF. The complexes were prepared in LR by manual mixing of BDNF with respective copolymers. Lanes 1 through 8 correspond to BDNF alone (lane 1), and Nano-BDNF prepared at various Z_−/+_: 0.1 (lane 2), 0.25 (lane 3), 0.50 (lane 4), 0.75 (lane 5), 1.0 (lane 6), 5.0 (lane 7), 10.0 (lane 8). The protein bands were visualized by Coomassie blue staining.

After optimizing the formulation of Nano-BDNF PSR-PLE and Nano-BDNF B5, we visualized their formation and migration through an agarose gel matrix. Jiang et al. [11] demonstrated that Nano-BDNF PEG-PLE, formulated at excess of polyanion, yielded complexes with a negative overcharge that migrated BDNF towards the cathode despite the protein’s intrinsic net positive charge. Nano-BDNF formulated with PSR-PLE and B5 behaved similarly, such that little or no migration was observed at Z_−/+_ < 5, whereas higher Z_−/+_ (Z_−/+_ = 5, 10) formulations yielded distinct bands migrating towards cathode (Figure 7). Notably, as in Jiang et al. [11], the free BDNF or Nano-BDNF complexes at Z_−/+_ < 1 remained in the wells with little apparent movement towards the anode. We believe that the HAGE approach is unable to distinguish the free protein and complexes formed at deficiency of the copolymer, because it is known that BDNF can bind to agarose and is also prone to aggregation [49]. In the complexes formed at lower mixing ratio, the BDNF molecule may not be completely masked by the bound copolymer and partially exposed. On the other hand, the mobility of the complexes formed at higher Z_−/+_ may indicate not only the overcharging but also complete masking of the BDNF by the copolymer that inhibits the aggregation and/or binding to agarose.

### 3.5. Zeta-Potential Characterization of Nano-BDNF

**Figure 8:**
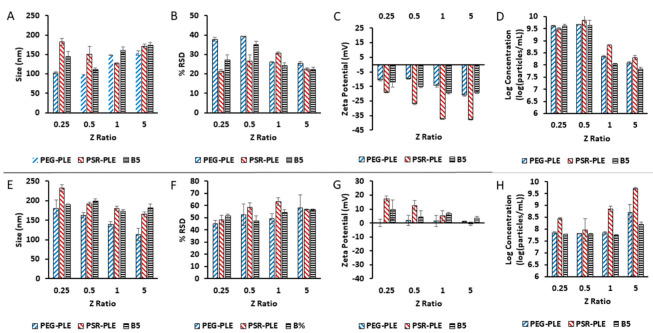
Particle characterization by (**A**,**E**) size, (**B**,**F**) dispersity, (**C**,**G**) zeta potential, and (**D**,**H**) concentration. Nano-BDNF was formulated in (**A**–**D**) 10 mM sodium phosphate, pH 7.4 and (**E**,**F**) 10 mM sodium acetate, pH 5.0 at room temperature using PEG-PLE, PSR-PLE, or B5. Values are mean ± SEM.

As an unambiguous method for characterization of Nano-BDNF complexes at both low and high mixing ratios, we used the NTA Zetaview machine, which directly measures the particle size and zeta potential in the same samples, and additionally reports the calculated dispersity as well as concentration of the particles (Figure 8). For this study, we used three block copolymers, PEG-PLE, PSR-PLE, and B5, and prepared the PICs by the microfluidic mixing. The overall pattern of behavior of the complexes in 10 mM sodium phosphate, pH 7.4 was very similar for all three copolymers. We were able to observe the formation of the nanosized complexes at Z_−/+_ as low as 0.25 (Figure 8A,B). These complexes were already net negatively charged at low Z_−/+_ ratio but increased in the absolute value of the charge with the further addition of anionic copolymer (Figure 8C). For all three copolymers, as the Z_−/+_ increased above 0.5, the concentration of the particles sharply decreased by over 10 times (with no precipitation observed) (Figure 8D). This phenomenon could be explained by the increased binding of the copolymer to BDNF upon increase of Z_−/+_, which promotes self-assembly of PICs with higher molecular masses and higher aggregation numbers of BDNF and copolymer. In contrast, at low Z_−/+_, we may be observing the formation of nascent complexes which have lower molecular masses and aggregation numbers. This assumption is only partially supported by the particle size measurements (Figure 8A). While there was a distinct trend for increases in the sizes of Nano-BDNF PEG-PLE and B5, no increases in the particle size were apparent in the case of PSR-PLE. However, the discussed transitions in the PICs may be masked by the shape effects which are not accounted for by our size measurement technique.

The self-assembly and overcharging effects in nano-BDNF must be dependent on the degree of ionization of the reacting anionic block copolymer. To test this, we conducted the same experiment at pH 5.0 (Figure 8E–H) using a 10 mM sodium acetate buffer, where the polyanions have lower ionization degree (Supplementary Figure S17, Table S1). In this case, the complexes formed at Z_−/+_ = 0.25 appeared to have positive charge, which gradually decreased as the amount of the anionic copolymer increased (Figure 8G). Interestingly, there was an increase in the concentration of the nanoparticles, most distinct in the cases of PEG-PLE and PSR-PLE, which suggests transition to PICs with lower molecular mass and aggregation numbers, i.e., opposite to what is seen at pH 7.4.

### 3.6. Thermodynamic Analysis of BDNF-Polyanion Association

**Figure 9:**
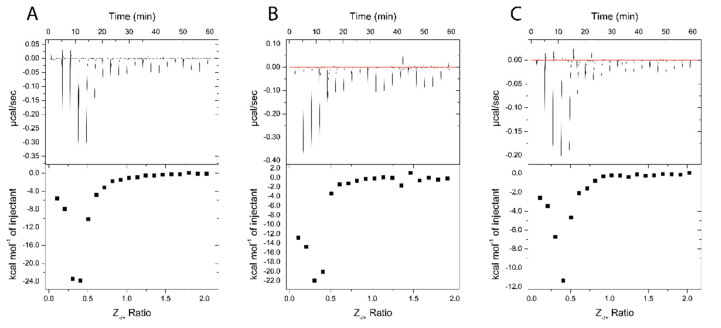
ITC thermograms of Nano-BDNF formation in 10 mM sodium phosphate buffer, pH 7.4. The holding cell was filled with 200 μL of 10 μM BDNF at 25 °C. (**A**) 88 μM PEG-PLE, (**B**) 88 μM PSR-PLE, and (**C**) 162 μM B5 were injected in 2 μL increments with a 3 min dwell time between each injection. Top: time dependence of the heat supplied to the sample cell for each injection of the block copolymer into the BDNF solution. Bottom: integrated ITC curves for the block copolymer binding with BDNF as a function of the charge ratio Z_−/+_.

To further investigate the phenomena observed in the previous experiment, we used ITC to measure the heat released as we added copolymer to BDNF. Our experiments were designed to keep the BDNF concentration constant as the anionic block copolymer increased step by step. The copolymer concentrations were adjusted to keep the number of polyacid repeating units uniform across different copolymers. Thus, the x-axis in our thermograms correlates to the Z_−/+_ ratio rather than the molar ratio. For clarity, the Z_−/+_ ratio equal to 1 corresponds to the polyanion/BDNF molar ratio 0.88 for both PEG-PLE and PSR-PLE and 1.63 for B5 due to the differences in the degrees of polymerization of the polyanion blocks of these copolymers (Figure 2). The addition of each copolymer dropwise to a well containing BDNF yielded exothermic reactions (Figure 9). Notably, none of the integrated ITC curves had a simple sigmoidal shape that would be expected for a simple single set of identical sites (SSIS) model for the molecules binding [50]. In fact, for each of the copolymers, there was a sharp minimum observed at Z_−/+_ between 0.25 and 0.5. The heat effects level off to the baseline at Z_−/+_ between 0.5 and 0.8. Notably, the pattern of heat release is similar to what might be expected of multiple ligands binding a transition metal [50]. In our system, this curve might be due to initial formation of nascent PICs formed at low Z_−/+_, which further self-assemble into mature Nano-BDNF nanoparticle as more block copolymer is added.

**Figure 10:**
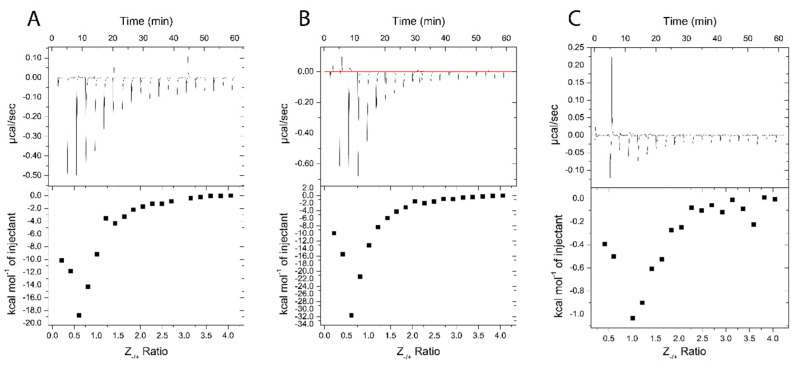
ITC thermograms of Nano-BDNF formation in 10 mM sodium acetate buffer, pH 5.0. The holding cell was filled with 200 μL of 10 μM BDNF in at 25 °C. (**A**) 88 μM PEG-PLE, (**B**) 88 μM PSR-PLE, and (**C**) 162 μM B5 were injected in 2 μL increments with a 3 min dwell time between each injection. Top: time dependence of the heat supplied to the sample cell for each injection of the block copolymer into the BDNF solution. Bottom: integrated ITC curves for the block copolymer binding with BDNF as a function of the charge ratio Z_−/+_.

To further validate the relationship between these processes and the charge ratio of the reacting components in the mixture, we again decreased the pH value of the system, keeping all other parameters the same. Although the overall shape of the curves did not change, at pH 5.0, the minimum in integrated ITC curves shifted to higher Z_−/+_ between 0.5 and 0.8 and the saturation region to Z_−/+_ between 1.5 and 2.0 (Figure 10, see also Supplementary Table S2 for comparison of plot features at different pH). The polyanionic blocks (PLE and PPaOx) in our block copolymers undergo ionization in the range from pH 4 to pH 8 (Supplementary Figure S17, Table S1). The shift of the position of the minimum in integrated ITC curves is consistent with a decrease in the amount of ionized carboxylic groups in the reacting polyacids that takes place at lower pH, and is indicative of the electrostatic nature of the polyion coupling reactions in this Z_−/+_ range. Interestingly, at pH 5.8, no such shift in the integrated ITC curves minimum and level off regions was observed (Supplementary Figure S18), which is probably explained by cooperative stabilization of the PICs, as will be discussed below. The overall magnitude of the exothermic heat effects seen at the integrated ITC curves minima differed for block copolymers and were notably smaller for B5, for which the heat release dramatically decreased at lower pH (Supplementary Table S2).

### 3.7. Stimulation of the TrkB Pathway

**Figure 11:**
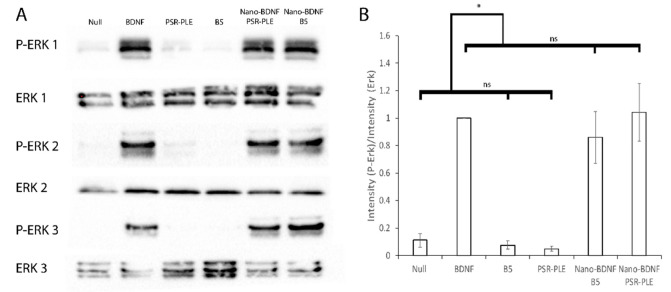
Stimulation of the TrkB Pathway by application of BDNF and Nano-BDNF formulations to NIH 3T3 TrkB cells. For each experiment (1–3), cultured cells were stimulated with 500 ng/mL of BDNF, equivalent amount of nano-BDNF formulated in media at Z_−/+_ = 5, or polymer alone. (**A**) Erk or P-Erk was detected using α-Erk or α-P-Erk via western blot. (**B**) The adjusted total lane volume (measured in signal intensity) of each lane on the blots was determined; then, the ratio of P-ErK/Erk was calculated. All values were normalized to the BDNF sample and averaged. Experiments were done in triplicate from the point of cell culture. Values are mean ± SEM, * *p* < 0.05. Statistical significance was established using an unpaired two-tailed Student’s *t*-test.

The potential therapeutic application of BDNF is premised on the effective stimulation of the TrkB kinase pathway which effects neuropotentiation, neuron survival, and repair [14,43]. Nanoformulation entails the possibility of making BDNF unavailable to the receptor TrkB. We applied Nano-BDNF to NIH 3T3 cells stably transfected with TrkB and measured the phosphorylation of a downstream kinase, extracellular signal-regulated kinase 1/2 (Erk). BDNF, Nano-BDNF B5 Z_−/+_= 2, and Nano-BDNF PSR-PLE Z_−/+_ = 5 stimulated the phosphorylation of Erk compared to the total Erk present in the cell (Figure 11). The availability of BDNF was acceptable and indicates that neither of our novel Nano-BDNF formulations inhibits TrkB signaling.

## 4. Discussion

The well-established immunogenicity of PEG and the high frequency of endogenous α-PEG antibodies indicate a dire need for alternative drug encapsulation technologies with comparable or lower immunogenic profiles [3,4,5,6,7]. A recent study identified anaphylaxis caused by the COVID-19 vaccines, which was disproportionally prevalent in women, most likely influenced by higher exposure in household products containing PEG [8,9]. Both poly(sarcosine) and poly(methyl-2-oxazolines) have demonstrated physical, protective, and stealth characteristics similar to PEG [19,20,21,26,27,31,39,42]. Critically, these alternative polymers do not currently have such extensive applications across several industries, which limits exposure for most of the population. Both poly(sarcosine) and poly(oxazolines) are relatively simple to manufacture and functionalize or are commercially available. Poly (oxazolines) in particular are a broad class of molecules that allow for hydrophobic, charged, and otherwise reactive moieties to be included in polymer structures [19,30,33,51]. Other work has taken advantage of the variety of poly(oxazoline) functional groups, including the stealth portion of the nanoparticles [30,31,33,37,44,52]. Additionally, LCROP allows excellent control over the length and order of blocks which are easily assessed using ^1^H NMR to determine structure as demonstrated in the results section.

The original Nano-BDNF formulation using PEG-PLE, described in Jiang et al. and Harris et al., demonstrated promising effects in disease models [10,11]. Indeed, the simple self-assembly made similar nanoparticles especially persuasive to attempt to reformulate. Our motivation was not necessarily to improve upon the prior PEG-PLE-based composition, but rather to explore and provide reasonable alternatives in which PEG is substituted by a similarly non-fouling and biocompatible polymer. Our candidate molecules with non-PEG protective blocks were suggested by their similarity to original formulation, especially in the context of the associative block. A winnowing strategy eliminated the polymers which yielded poorly formed or inconsistent particles and we found that optimal particles were formed in the same buffer as Nano-BDNF PEG-PLE.

We implemented microfluidic mixing throughout our study to prepare the complexes. The small-scale batch formulation by manual mixing carries the inherent drawback of batch-by-batch variation due to non-uniform mixing. We overcome this limitation by extensive use of microfluidic mixing, which, in addition to improving reproducibility, decreases size and narrows dispersity by mixing the components in a controlled manner. Microfluidic mixing has become an increasingly common technique, especially in the formulation of lipid nanoparticles [48]. Furthermore, in our experience, the dispersity of PICs is improved through application of this technology and consistency across batches is also improved [11]. Here, our choice of a reusable, simple, cheap, in-house designed mixer indicates that even low-level implementation of these technologies can improve the consistency and quality of formulations.

Nanoformulation carries inherent risk of deactivation of an intended therapy, especially for signaling molecules which must access and bind to a target receptor. Here, we demonstrate that despite inclusion of the BDNF into the PICs at the excess of the copolymers, both our re-formulated Nano-BDNF variants remain active with respect to TrkB signaling pathway in cells. This has been previously reported by us for Nano-BDNF PEG-PLE and the mechanism was demonstrated [11]. Specifically, we have previously shown that while the complexes remain stable in the presence of salt and serum proteins (human serum albumin, IgG, secreted IgA), the TrkB receptor can readily abstract the BDNF molecule, presumably due to higher affinity to the BDNF active site compared to the anionic block copolymer. In this case, TrkB can access and bind BDNF, which yields a signaling cascade that we monitor via Erk phosphorylation. We believe that we observe activation of the receptor with the reformulated Nano-BDNF particles that proceeds via similar mechanism as was reported before [11]. Moreover, based on prior molecular modeling analysis [11], the polyanion binds to the same active site as the TrkB, thereby competitively masking this site in the absence of the specific receptor. Therefore, this class of PICs is particularly well suited for delivery of BDNF and possibly other neurotrophins. Non-covalent interactions allow some reorganization and may enable a receptor with strong, specific binding to access the signaling molecule. Conversely, the preferential binding with the anionic blocks of the copolymers protects from opsonization [10,11].

Our study also provides new insight on the complexation of the BDNF with anionic block copolymers. Using the highly efficient, multi-utility nanoparticle tracking technique, we confirm the formation of the PICs at the deficiency of the anionic block copolymers. Based on the patterns of the particle zeta potential and concentration, we propose that these complexes form initially as the nascent species that reorganize into mature Nano-BDNF particles as the amounts of the added anionic block copolymers (Z_−/+_ ratio) increase. An independent ITC experiment confirms the distinct processes at lower and higher Z_−/+_ ratios. The processes of polymer coupling at the block copolymer deficiency at lower Z_−/+_ are strongly exothermic, which results in the appearance of the deep minima in the integrated curve. This exothermic effect vanished as the amount of the block copolymer increased at higher Z_−/+_. The data from our ITC experiments were not easily fit by SSIS models or a two-step model such as DNA condensation [50,53,54]. However, the ITC pattern observed for complexation of net cationic BDNF with our polyanions appears to qualitatively mirror that previously observed for the binding of DNA with polycations as reported by us and Kataoka [53,55].

There are, however, notable differences. First, our systems display strongly exothermic effects, while the DNA and polycation binding is endothermic. Second, the magnitude of our effect, although varying for different polyanions, is at least one order of magnitude greater than the effects observed for DNA and polycation binding. The small enthalpic effects observed for DNA and polycation binding are typical for entropy-driven processes due to the counterion release [55], and could also be compounded by the conformational transitions due to the DNA condensation [53]. In our case, BDNF displays 44 unique, positively charged Lys and Arg residues on its exterior and based on the prior molecular modeling binding of the polycarboxylic acid to it involves formation of multiple hydrogen bonds [11]. The hydrogen bond formation is an exothermic process which is consistent with our observation [56].

Unsurprisingly, the complexation is dependent on pH, which alters ionization of the polyacid. In the Supplementary Materials, we provided the titration results and ionization characteristics of all three anionic block copolymer used in our work. As we decrease the pH to 5.0, the degree of ionization decreases dramatically, and the characteristics of the complexes change. By using the nanoparticle tracking technique, we observe formation of positively charged species at low amounts of the added anionic block copolymers (Z_−/+_ ratio). We also observe changes in the ITC patterns where the amount of the block copolymer required in the exothermic binding increases. The greatest effects appear with the poly(oxazoline) block copolymer B5, which has the highest value of pK_a_. This suggests a strong effect of the polyanion ionization in the formation of Nano-BDNF complexes. However, we would like to point out that the direct quantitative relation of the degree of ionization of the weak polyelectrolytes to polyion complexation should be done with caution because of the cooperative stabilization of the ionized forms [57]. Specifically, in the discussed pH range the BDNF molecule is positively charged, and binding to it can stabilize the ionized forms of the polycarboxylic acids even at low pH (a phenomenon we cannot demonstrate here by potentiometric titration because of prohibitive costs). This may explain why we do not see much change in the ITC results at pH 5.8. Most importantly, our current findings suggest that by carefully selecting pH during the manufacturing processes, the properties of polyelectolyte formulations of neurotrophins can be optimized. Overall, deeper understanding of the physics which dictate nanoparticle formation and how varying conditions affect formulation can yield valuable insight to how future polyelectrolyte complex formulations are designed or reformulated.

## 5. Conclusions

We demonstrate the suitability of two novel polymers to substitute for PEG-PLE in reformulations of Nano-BDNF that are free of PEG. Particles of similar size and properties are formed through simple, microfluidic mixing. Overall, we demonstrate that by preserving the specific attributes of the associative and protective blocks, we can produce nanoparticles that behave in a similar manner. The new results are instrumental for the design of future formulations of neurotrophins for clinical use.

## Data Availability

Data are available from authors upon reasonable request.

## Supplementary Materials

The following supporting information can be downloaded at: https://www.mdpi.com/article/10.3390/pharmaceutics14071391/s1, Figure S1. Schematics showing configuration and dimensions of the mixer used for microflu-idics formulation; Figure S2. 1H NMR spectra of the four PMeOx-PPaOx-PMeOx polymers; Figure S3. Intensity weighted particle size and dispersity of Nano-BDNF PSR-PLE; Figure S4. Number weighted particle size and kilocount per second of Nano-BDNF PSR-PLE measurements in DLS of the same samples as shown Figure S3; Figure S5. Intensity weighted particle size and dispersity of Nano-BDNF B1; Figure S6. Number weighted particle size and kilocount per second of Nano-BDNF B1 meas-urements in DLS of the same samples as shown Figure S5; Figure S7. Intensity weighted particle size and dispersity of Nano-BDNF B2; Figure S8. Number weighted particle size and kilocount per second of Nano-BDNF B2 meas-urements in DLS of the same samples as shown Figure S7; Figure S9. Intensity weighted particle size and dispersity of Nano-BDNF B3; Figure S10. Number weighted particle size and kilocount per second of Nano-BDNF B3 measurements in DLS of the same samples as shown Figure S9; Figure S11. Intensity weighted particle size and dispersity of Nano-BDNF B5; Figure S12. Number weighted particle size and kilocount per second of Nano-BDNF B5 measurements in DLS of the same samples as shown Figure S11; Figure S13. Intensity weighted particle size and dispersity of Nano-BDNF in the presence of small-molecular mass electrolyte; Figure S14. Number weighted particle size and kilocount of Nano-BDNF in the presence of small-molecular mass electrolyte measured by DLS of the same samples as shown Figure S13; Figure S15. Representative transmission electron microscopy (TEM) images of mixer formu-lated Nano-BDNF (Z_−/+_ = 5) in 10 mM phosphate, pH 7.4; Figure S16: Particle size and distribution of mixer formulated Nano-BDNF in (A,C) PBS and (B,D) LR solution over time; Figure S17. Titration of Block Copolymers; Table S1. The calculated effective pKa values and degrees of ionization at critical pH values; Figure S18. ITC thermograms of Nano-BDNF formation in 10 mM sodium phosphate buffer, pH 5.8; Table S2. Critical Z_−/+_ values of in the analysis of ITC thermograms; Figure S19. Particle size and distribution of mixer formulated Nano-BDNF in DMEM. Refs. [58,59] are cited in Supplementary Materials file.
